# GluA1 subunit ubiquitination mediates amyloid-β-induced loss of surface α-amino-3-hydroxy-5-methyl-4-isoxazolepropionic acid (AMPA) receptors

**DOI:** 10.1074/jbc.M116.774554

**Published:** 2017-04-04

**Authors:** Sumasri Guntupalli, Se Eun Jang, Tianyi Zhu, Richard L. Huganir, Jocelyn Widagdo, Victor Anggono

**Affiliations:** From the ‡ Clem Jones Centre for Ageing Dementia Research, Queensland Brain Institute, The University of Queensland, Brisbane, Queensland 4072, Australia and; the § Department of Neuroscience and Kavli Neuroscience Discovery Institute, The Johns Hopkins University School of Medicine, Baltimore, Maryland 21205

**Keywords:** α-amino-3-hydroxy-5-methyl-4-isoxazolepropionic acid receptor (AMPA receptor, AMPAR), amyloid-β (Aβ), phosphorylation, trafficking, ubiquitylation (ubiquitination)

## Abstract

The accumulation of soluble amyloid-β (Aβ) peptides produces profound neuronal changes in the brain during the pathogenesis of Alzheimer's disease. Excessive levels of Aβ disrupt excitatory synaptic transmission by promoting the removal of synaptic AMPA receptors (AMPARs), dendritic spine loss, and synaptic depression. Recently, activity-dependent ubiquitination of the GluA1 subunit has been shown to regulate the intracellular sorting of AMPARs toward late endosomes for degradation. However, whether this ubiquitin signaling pathway mediates Aβ-induced loss of surface AMPARs is unknown. In this study, we demonstrate that acute exposure of cultured neurons to soluble Aβ oligomers induces AMPAR ubiquitination concomitant with the removal of AMPARs from the plasma membrane. Importantly, expression of the GluA1 ubiquitin-deficient mutants inhibited the adverse effects of Aβ on the surface expression of AMPARs in neurons. Furthermore, we revealed the cross-talk between GluA1 ubiquitination and phosphorylation, in particular phosphorylation at Ser-845, which is crucial for AMPAR recycling and is known to be dephosphorylated in the presence of Aβ. Our data showed that the GluA1 ubiquitin-deficient mutant enhances GluA1 phosphorylation on Ser-845. Conversely, the GluA1 S845D phosphomimetic mutant reduced binding with Nedd4-1 and hence the ubiquitination of AMPARs. Importantly, the GluA1 S845D mutant also prevented Aβ-induced removal of surface AMPARs. Taken together, these findings provide the first demonstration of the dynamic cross-modulation of GluA1 ubiquitination and phosphorylation, a process that is perturbed by Aβ, in regulating the membrane sorting decision that ultimately determines the number of AMPARs on the cell surface.

## Introduction

Memory loss and progressive decline of higher cognitive functions are common clinical features of Alzheimer's disease. Accumulating evidence from human genetics, transgenic mouse models, and biochemical studies has indicated a role for amyloid-β (Aβ)^4^ peptides in the etiology and pathogenesis of this disease (1). Studies have shown that soluble oligomeric forms of Aβ peptides derived by proteolytic cleavage of amyloid precursor protein (APP), of which Aβ(1–42) (hereafter referred to as Aβ) is one of the predominant species, exert strong detrimental effects on the structure and function of synapses that could be directly linked to the learning and memory deficits in Alzheimer's patients (2, 3). At the cellular level, there is strong evidence that Aβ is linked to a range of detrimental neurobiological processes, including reduced excitatory synaptic transmission, loss of dendritic spines, aberrant neuronal network activity, inflammation, and excitotoxic neuronal death (2, 4, 5).

Glutamate receptors are particularly vulnerable to the neurotoxic effect of Aβ (6). Congruent with the hypothesis that Aβ causes synaptic failure (1), various transgenic mice carrying APP mutations linked to familial or autosomal dominant forms of Alzheimer's disease exhibit dendritic spine loss, deficits in glutamatergic synaptic transmission, and impaired learning prior to the development of plaques and neuronal death (7). Furthermore, application of Aβ potently inhibits hippocampal long-term potentiation both *in vitro* and *in vivo* due to synaptic depression of excitatory neurotransmission (8, 9). Indeed, elevated Aβ levels have been shown to induce the removal of AMPA receptors (AMPARs), the major glutamate receptors that mediate fast excitatory neurotransmission in the mammalian central nervous system (6, 10–13). Synaptic removal of AMPARs is necessary and sufficient to produce the subsequent loss of dendritic spines and synaptic depression (10). More importantly, the loss of AMPARs has also been observed in post-mortem Alzheimer's disease brains (14, 15). Although some studies have proposed that Aβ may hijack the normal physiological process of AMPAR trafficking to produce synaptic depression (6, 10), the molecular mechanisms underlying the Aβ-induced loss of synaptic AMPARs remain elusive.

It has been proposed that an Aβ-induced decrease in surface AMPARs is mediated in part by misregulation of AMPAR post-translational modifications, which are known to control multiple aspects of AMPAR trafficking (16). For example, soluble oligomeric Aβ induces dephosphorylation of GluA1 at Ser-845 (11) as well as enhances AMPA-induced ubiquitination of GluA1 (17). Phosphorylation of GluA1 at Ser-845 by protein kinase A (PKA) modulates the probability of channel opening (18) and promotes AMPAR recycling (19, 20), whereas ubiquitination of GluA1 at Lys-868 by the E3 ligase Nedd4-1 regulates the intracellular sorting and degradation of AMPARs (21–23). Collectively, these data suggest that Aβ promotes the internalization and degradation of AMPARs as well as negatively regulates the recycling of these receptors back to the plasma membrane. Interestingly, a recent study has shown that shRNA-mediated knockdown of Nedd4-1 blocks the Aβ-induced reduction in surface AMPARs (17); however, whether this process is mediated by direct ubiquitination of AMPARs is unclear. Furthermore, it is also unknown whether Aβ-induced dephosphorylation of GluA1 at Ser-845 affects the ubiquitination of AMPARs.

In the present study, we reveal that direct ubiquitination of the GluA1 subunit of AMPARs is an essential signaling pathway that mediates the Aβ-induced loss of surface AMPARs in primary cortical neurons. In addition, we show that the GluA1 S845D phosphomimetic mutant negatively regulates the ubiquitination of AMPARs due to reduced binding of Nedd4-1. This in turn also inhibits the loss of surface AMPAR expression following Aβ treatment. These results provide insights into the importance of dynamic cross-talk between GluA1 phosphorylation and ubiquitination in controlling AMPAR trafficking and cell surface expression.

## Results

### Aβ induces ubiquitination of AMPARs

**Figure 1.**
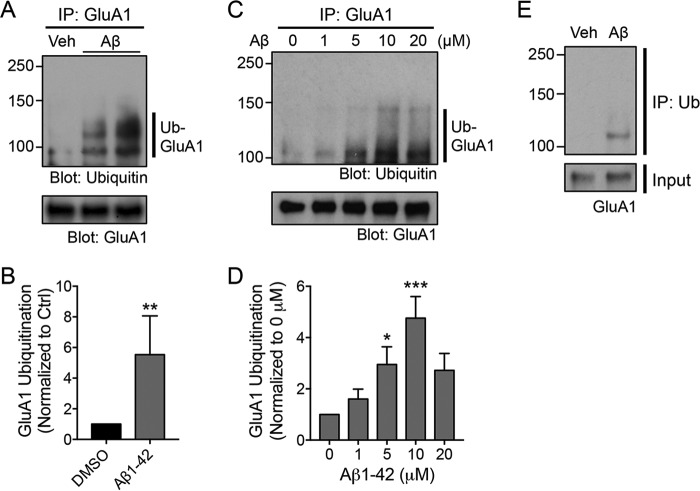
**Aβ induces the ubiquitination of the GluA1 subunit of AMPARs in primary neurons.** *A*, cultured neurons were incubated with DMSO (*Veh*, vehicle control) or 5 μm Aβ for 1 h and immediately lysed in 1% SDS. Diluted lysates were immunoprecipitated with anti-GluA1 antibodies (*IP: GluA1*). Eluted proteins were subjected to Western blot analysis and probed with anti-ubiquitin and anti-GluA1 antibodies. Ubiquitinated GluA1 is indicated by the *vertical bar* (*Ub-GluA1*). The *two right-hand lanes* represent biological replicates. *B*, the effect of Aβ treatment on GluA1 ubiquitination was quantified and normalized to a DMSO control (*Ctrl*). Data are represented as the mean of five independent experiments (Mann-Whitney test; **, *p* < 0.01; *n* = 5). *C*, cultured neurons were incubated with increasing concentrations of Aβ for 1 h and subjected to the ubiquitination assay. *D*, a dose-response curve of Aβ effects on the ubiquitination of GluA1. Data are represented as the mean of four independent experiments (one-way ANOVA; *, *p* < 0.05; ***, *p* < 0.001; *n* = 4). *E*, representative immunoblots depicting Aβ-induced ubiquitination of the GluA1 subunit of AMPARs following immunoprecipitation assays using anti-ubiquitin antibodies (*IP: Ub*) (from two independent experiments). *Error bars* represent S.E.

It is well established that soluble Aβ oligomers, either synthetic or those secreted from cell lines, potently block hippocampal long-term potentiation and promote long-term depression via mechanisms that involve the down-regulation of surface AMPARs (9, 10, 17). Recent studies have demonstrated a role for GluA1 ubiquitination in regulating the internalization and intracellular sorting of AMPARs toward late endosomes (21–23). Based on these findings, we hypothesized that the same ubiquitin signaling pathway is involved in the Aβ-induced reduction of surface AMPAR expression in neurons. To investigate this, we first examined the effect of soluble synthetic Aβ oligomers on AMPAR ubiquitination in cultured cortical neurons by immunoprecipitating the GluA1 subunit from lysates of neurons that had been treated with 5 μm Aβ for 1 h. We found that GluA1 was robustly ubiquitinated following Aβ treatment (5.53 ± 2.5-fold of DMSO-treated control) (Fig. 1, *A* and *B*). Next, we treated neurons with different concentration of Aβ for 1 h and generated a dose-response curve. We found that Aβ enhanced the ubiquitination of GluA1 in a dose-dependent manner, producing the maximal effect at 10 μm (4.8 ± 0.8-fold of DMSO-treated control) (Fig. 1, *C* and *D*). However, we chose to use 5 μm for the remainder of our study because this was the minimal concentration of Aβ that significantly induced GluA1 ubiquitination (3.0 ± 0.7-fold of DMSO-treated control) (Fig. 1, *C* and *D*), thereby minimizing Aβ neurotoxicity that might lead to cell death. To confirm these findings, we performed a reverse immunoprecipitation assay using anti-ubiquitin antibodies on lysates extracted from Aβ-treated neurons and then blotted them with specific antibodies against the GluA1 subunit. We observed high-molecular-weight GluA1 immunoreactivity in the lysates of Aβ-treated neurons that was absent in the control lane (Fig. 1*E*). Together, these results demonstrate that Aβ induces the ubiquitination of the GluA1 subunit of AMPARs in primary cortical neurons.

### GluA1 ubiquitination mediates Aβ-induced down-regulation of surface AMPARs

**Figure 2.**
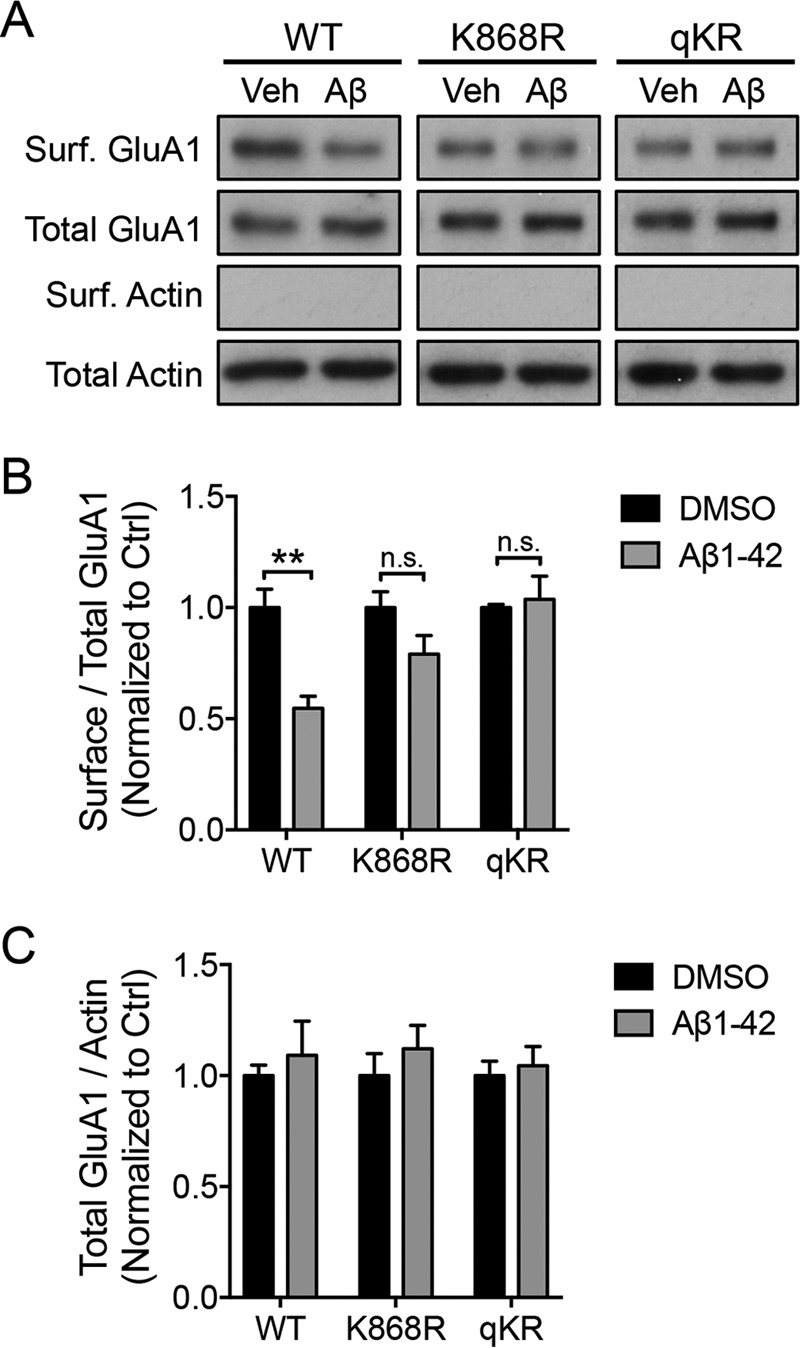
**GluA1 ubiquitination is necessary for the Aβ-induced reduction of surface AMPARs.** *A*, cortical neurons were electroporated with pH-GluA1 constructs, either WT or ubiquitin-deficient mutants as indicated, prior to plating. qKR contains quadruple mutations of Lys-813, Lys-819, Lys-822, and Lys-868 in the GluA1 C-terminal tail into arginines. At DIV14, neurons were treated with 5 μm Aβ or DMSO for 1 h and subjected to a surface (*Surf.*) biotinylation assay prior to cell lysis. Neuronal lysates were then incubated with NeutrAvidin beads to purify surface receptors. Eluted proteins and total cell lysates were subjected to Western blot analysis and probed with anti-GluA1 and anti-β-actin antibodies. The effects of Aβ on the levels of surface (*B*) and total (*C*) receptor expression were quantified as surface/total receptor ratios and as total receptor/β-actin ratios, respectively, and normalized to DMSO controls (*Ctrl*). Data represent the mean of three independent experiments (Mann-Whitney test; **, *p* < 0.01; *n.s.*, not significant; *n* = 5). *Error bars* represent S.E. *Veh*, vehicle.

We next examined the loss of surface AMPARs following 1 h of Aβ treatment. Cortical neurons were electroporated with pHluorin (pH-sensitive GFP)-tagged GluA1 (pH-GluA1), either wild type, K868R, or qKR (all four lysines in the C-terminal tail mutated to arginines; K813R/K819R/K822R/K868R) at days *in vitro* (DIV) 0 and were subjected to a biotinylation assay to label the surface proteins at DIV13. As expected, 1-h incubation with Aβ caused a robust down-regulation of surface pH-GluA1 expression (GluA1 wild type (WT), 54.7 ± 5.4% of DMSO-treated control) (Fig. 2, *A* and *B*). Interestingly, Aβ failed to reduce the surface expression of both the pH-GluA1 ubiquitin-deficient mutants (GluA1 K868R, 79.1 ± 8.4%; GluA1 qKR, 103.7 ± 10.5% of DMSO-treated controls) (Fig. 2, *A* and *B*). No apparent alteration in the expression of total pH-GluA1 was observed after Aβ treatment across genotypes (Fig. 2, *A* and *C*).

**Figure 3.**
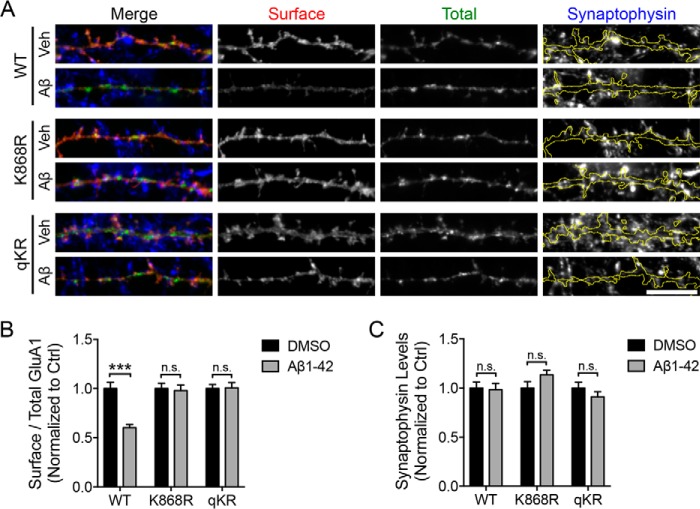
**GluA1 ubiquitination is necessary for the Aβ-induced reduction of surface AMPARs.** *A*, cortical neurons were transfected with pH-GluA1 constructs, either WT or ubiquitin-deficient mutants as indicated, at DIV12. qKR contains quadruple mutations of Lys-813, Lys-819, Lys-822, and Lys-868 in the GluA1 C-terminal tail into arginines. At DIV14, neurons were treated with 5 μm Aβ or DMSO (*Veh*) for 1 h and incubated with anti-GFP antibodies prior to fixation to visualize surface pH-GluA1 expression (*red*). Total pH-GluA1 expression was determined with the endogenous GFP signal (*green*), whereas the level of synaptophysin was visualized by immunostaining with anti-synaptophysin antibodies (*blue*). *Scale bar*, 10 μm. The effects of Aβ on the levels of surface receptor expression (*B*) and synaptophysin (*C*) were quantified as surface/total receptor ratios and as total synaptophysin staining per dendritic area, respectively, and normalized to DMSO controls (*Ctrl*). Data represent the mean of two independent experiments (Mann-Whitney test; ***, *p* < 0.001; *n.s.*, not significant; *n* = 30). *Error bars* represent S.E.

To confirm this finding, we performed a surface staining assay on neurons expressing pH-GluA1 (either wild type or K868R or qKR mutant) with anti-GFP antibodies that recognize the extracellular pHluorin. Consistent with our surface biotinylation data, Aβ robustly reduced the surface expression of wild-type pH-GluA1 (GluA1 WT, 59.3 ± 3.2% of DMSO-treated control) (Fig. 3, *A* and *B*). In contrast, Aβ failed to reduce the surface expression of both the pH-GluA1 ubiquitin-deficient mutants (GluA1 K868R, 95.8 ± 5.5%; GluA1 qKR, 101.8 ± 5.3% of DMSO-treated controls) (Fig. 3, *A* and *B*). No apparent alteration in the expression of the presynaptic marker synaptophysin on the dendrite was observed after Aβ treatment across genotypes (Fig. 3, *A* and *C*). Collectively, these data provide the first evidence for the requirement of direct ubiquitination of GluA1 in mediating the ability of Aβ to promote the down-regulation of surface AMPARs in primary cortical neurons.

### The cross-talk between GluA1 phosphorylation and ubiquitination

**Figure 4.**
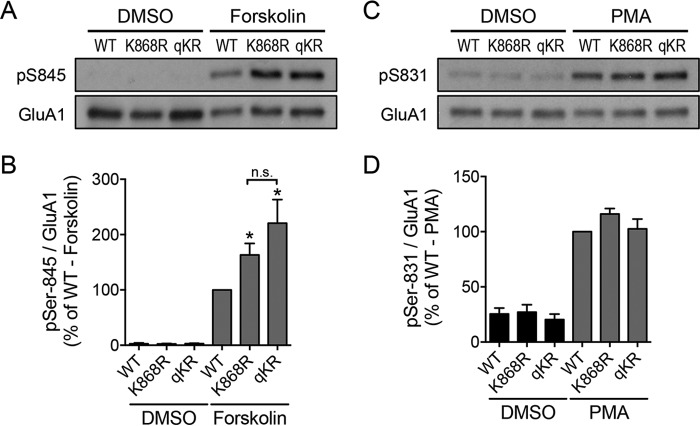
**Ubiquitination negatively regulates GluA1 phosphorylation at Ser-845.** Cortical neurons were electroporated with pH-GluA1 constructs, either WT or ubiquitin-deficient mutants as indicated, prior to plating. qKR contains quadruple mutations of Lys-813, Lys-819, Lys-822, and Lys-868 in the GluA1 C-terminal tail into arginines. At DIV14, neurons were treated with 20 μm forskolin (*A*), 0.1 μm PMA (*C*), or DMSO for 10 min and immediately lysed with 1× SDS sample buffer. Total cell lysates were subjected to Western blot analysis and probed with anti-GluA1 phospho-Ser-845, anti-GluA1 phospho-Ser-831, and anti-GluA1 antibodies. The effects of GluA1 ubiquitin-deficient mutants on the phosphorylation levels at Ser-845 (*B*) and Ser-831 (*D*) were quantified as phospho-/total receptor ratios and normalized to wild-type controls. Data represent the mean of five independent experiments (one-way ANOVA; *, *p* < 0.05; *n.s.*, not significant; *n* = 12). *Error bars* represent S.E.

The GluA1 subunit is phosphorylated at two major sites in its C-terminal domain, namely Ser-831 and Ser-845, by protein kinase C (PKC) or calcium/calmodulin-dependent kinase II and PKA, respectively (18, 24–27). Although phosphorylation of GluA1 is known to be important for AMPAR trafficking and synaptic plasticity (28), it has also been shown to prevent AMPARs from undergoing lysosomal degradation (29, 30). We proposed that the phosphorylation of GluA1 might modulate its ubiquitination status to fine-tune the trafficking and stability of AMPARs in neurons. To address this possibility, we first examined the effects of GluA1 ubiquitin-deficient mutants on Ser-831 and Ser-845 phosphorylation induced by phorbol ester PMA and forskolin treatment, respectively. We found that neurons that overexpressed pH-GluA1 K868R and qKR mutants displayed significantly enhanced levels of Ser-845 phosphorylation compared with pH-GluA1 wild type (GluA1 K868R, 163.1 ± 21%; GluA1 qKR, 220.7 ± 42.6% of forskolin-treated WT) (Fig. 4, *A* and *B*). These effects were specific for Ser-845 phosphorylation as the ubiquitin-deficient mutants did not alter the levels of Ser-831 phosphorylation induced by PMA (GluA1 K868R, 116.0 ± 4.9%; GluA1 qKR, 102.6 ± 8.9% of PMA-treated WT) (Fig. 4, *C* and *D*).

**Figure 5.**
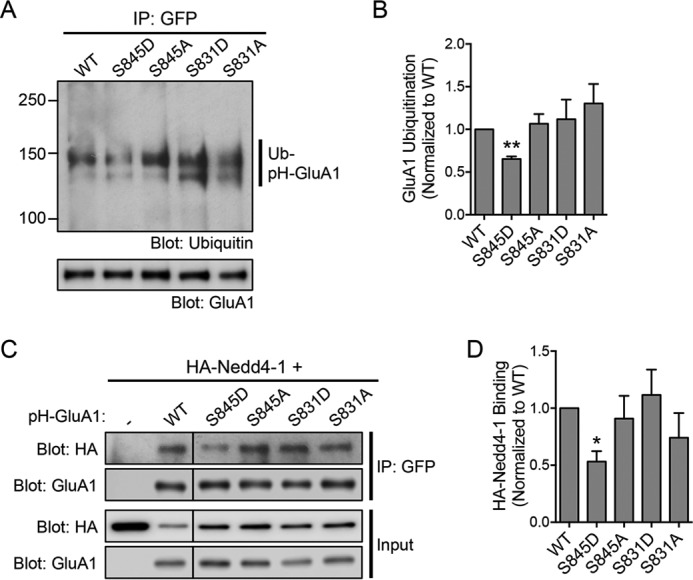
**The S845D phosphomimetic mutant blocks GluA1 ubiquitination by reducing its interaction with Nedd4-1.** *A*, cortical neurons were electroporated with pH-GluA1 constructs, either WT, phosphodeficient (alanine mutations), or phosphomimetic (aspartate mutations) as indicated, prior to plating. At DIV14, neurons were treated with 100 μm AMPA in the presence of 1 μm tetrodotoxin and 50 μm d-2-amino-5-phosphonovaleric acid for 10 min and immediately lysed in 1% SDS. Diluted lysates were immunoprecipitated with anti-GFP antibodies (I*P: GFP*). Eluted proteins were subjected to Western blot analysis and probed with anti-ubiquitin (*Ub*) and anti-GluA1 antibodies. *B*, the effects of GluA1 phosphorylation mutants on agonist-induced ubiquitination were quantified and normalized to the wild-type control. Data are represented as the mean of three independent experiments (one-way ANOVA; **, *p* < 0.01; *n* = 4). *C*, HEK293 cells were transfected with HA-Nedd4-1 alone (−; *lane 1*) or together with pH-GluA1 constructs, either wild type, phosphodeficient (alanine mutations), or phosphomimetic (aspartate mutations) as indicated. Cells were lysed 48 h later and subjected to immunoprecipitation assays with anti-GFP antibodies (*IP: GFP*). Total cell lysates and eluted proteins were subjected to Western blot analysis and probed with anti-HA and anti-GluA1 antibodies. *D*, the effects of GluA1 phosphorylation mutants on HA-Nedd4-1 binding were quantified and normalized to the wild-type control. Data are represented as the mean of three independent experiments (one-way ANOVA; *, *p* < 0.05; *n* = 3). *Error bars* represent S.E.

Next, we investigated the reciprocal effects of GluA1 phosphorylation mutants on the levels of AMPA-induced ubiquitination of this subunit. To do this, we electroporated pH-GluA1, either wild type, phosphodeficient mutant (Ser to Ala mutation), or phosphomimetic mutant (Ser to Asp mutation), at DIV0 and performed the ubiquitination assay at DIV13. We found that the pH-GluA1 S845D phosphomimetic mutant, but not the pH-GluA1 S845A phosphodeficient mutant, reduced the amount of GluA1 ubiquitination evoked by AMPA treatment (GluA1 S845D, 65.2 ± 3.1%; GluA1 S845A, 106.6 ± 11.4% of WT control) (Fig. 5, *A* and *B*). In contrast, neither the S831A nor the S831D mutant affected the level of GluA1 ubiquitination induced by AMPA treatment (GluA1 S831A, 130.2 ± 23.0%; GluA1 S831D, 111.9 ± 23.0% of WT control) (Fig. 5, *A* and *B*). These data indicate that the phosphorylation of Ser-845 negatively regulates GluA1 ubiquitination and that receptors that are not ubiquitinated have a higher level of Ser-845 phosphorylation.

To further understand the underlying mechanism, we investigated the effects of GluA1 phosphomutants on Nedd4-1 binding. HEK293 cells were co-transfected with HA-Nedd4-1 and pH-GluA1 constructs, either wild type or Ser-831/Ser-845 phosphodeficient and phosphomimetic mutants. As expected, wild-type pH-GluA1 interacted with HA-Nedd4-1 in the co-immunoprecipitation assay (Fig. 5, *C* and *D*). However, their interaction was significantly reduced only by the S845D substitution, whereas the other mutants had no effect on GluA1-Nedd4-1 binding (GluA1 S845D, 53.2 ± 9.1%; GluA1 S845A, 90.9 ± 19.9%; GluA1 S831D, 111.6 ± 22.2%; GluA1 S831A, 74.1 ± 21.6% of WT control) (Fig. 5, *C* and *D*). We also attempted to perform this experiment in cultured neurons, but we were unable to detect a specific interaction between pH-GluA1 and endogenous Nedd4-1. Collectively, these results suggest that the phosphorylation of GluA1 on Ser-845 is likely to inhibit its binding to Nedd4-1, thereby reducing the level of GluA1 ubiquitination.

### GluA1 S845D blocks Aβ-induced down-regulation of surface AMPARs

**Figure 6.**
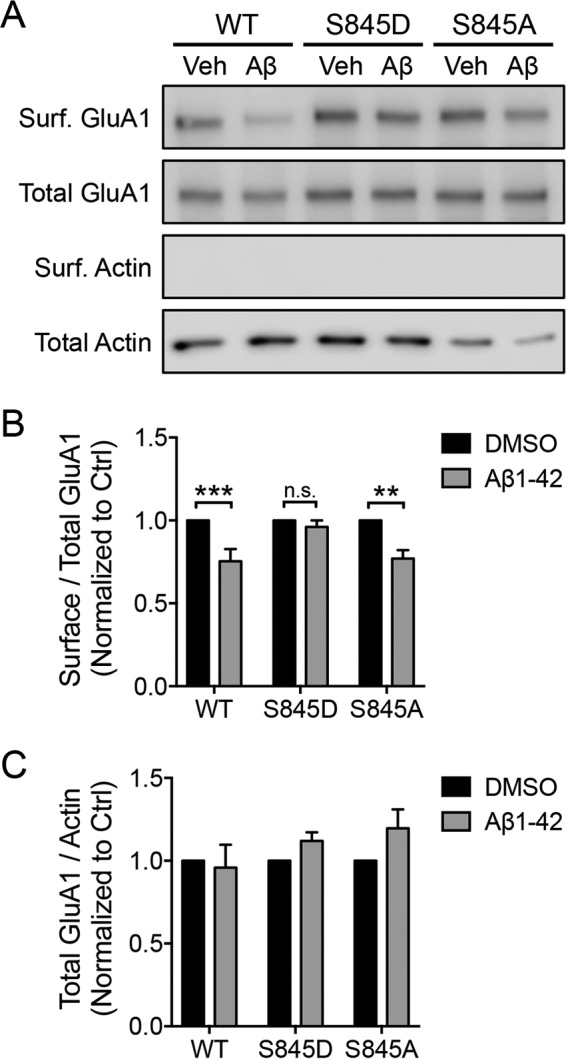
**The GluA1 S845D phosphomimetic mutant prevents the Aβ-induced reduction of surface AMPARs.** *A*, cortical neurons were electroporated with pH-GluA1 constructs, either wild type, phosphodeficient (alanine mutations), or phosphomimetic (aspartate mutations) as indicated, prior to plating. At DIV14, neurons were treated with 5 μm Aβ or DMSO for 1 h and subjected to the surface (*Surf.*) biotinylation assay prior to cell lysis. Neuronal lysates were then incubated with NeutrAvidin beads to purify surface receptors. Eluted proteins and total cell lysates were subjected to Western blot analysis and probed with anti-GluA1 and anti-β-actin antibodies. The effects of Aβ on the levels of surface (*B*) and total (*C*) receptor expression were quantified as surface/total receptor ratios and as total receptor/β-actin ratios, respectively, and normalized to DMSO controls (*Ctrl*). Data represent the mean of three independent experiments (Mann-Whitney test; **, *p* < 0.01; ***, *p* < 0.001; *n.s.*, not significant; *n* = 5). *Error bars* represent S.E. *Veh*, vehicle.

Given that a previous study has shown that an Aβ-induced reduction in surface GluA1 is linked to persistent dephosphorylation of GluA1 at Ser-845 (11), we considered that dephosphorylation of GluA1 at Ser-845 may be an upstream signal that allows GluA1 to be ubiquitinated and mediate the Aβ-induced reduction in surface AMPARs. Given that GluA1 S845D binds weakly to Nedd4-1 and has reduced levels of ubiquitination, it should prevent Aβ-induced down-regulation of surface AMPARs. To test this hypothesis, we examined the surface expression of pH-GluA1 S845D and S845A mutants following 1 h of Aβ treatment. When surface expression of pH-GluA1 was examined, the phosphomimetic S845D mutant inhibited the neurotoxic effect of Aβ, whereas the wild type and phosphodeficient S845A mutant had significantly lower expression on the plasma membrane (GluA1 WT, 75.3 ± 7.5%; GluA1 S845D, 96.1 ± 4.0%; S845A, 77.0 ± 5.1% of DMSO-treated controls) (Fig. 6, *A* and *B*). No apparent alteration in the expression of total pH-GluA1 expression was observed after Aβ treatment across genotypes (Fig. 6, *A* and *C*). These data indicate that the phosphorylation of GluA1 at Ser-845 is essential to recycle AMPARs back to the plasma membrane. Thus, under pathological conditions, Aβ-induced aberrant dephosphorylation of GluA1, due to an increase in calcineurin activity (11, 31), is likely to promote GluA1 ubiquitination and reroute the intracellular trafficking of GluA1-containing vesicles from recycling to late endosomes.

## Discussion

Dynamic trafficking of AMPARs between intracellular compartments and the plasma membrane underlies plastic changes in the synaptic strength of excitatory synapses in the brain (32). Synaptic depression of glutamatergic neurotransmission is one of the major consequences of acute Aβ toxicity, a mechanism that is believed to underlie synaptic and structural plasticity deficits in the pathophysiology of Alzheimer's disease (2, 4, 6). It is well established that Aβ-induced synaptic depression is driven primarily by the removal of surface AMPARs, which involves many signal transduction pathways that are also required for the induction of long-term depression (6). Molecular and pharmacological interventions that inhibit the internalization of AMPARs are able to rescue Aβ-induced synaptic deficits *in vitro* and cognitive impairments in an Alzheimer's mouse model (10, 31, 33, 34). These data support the rationale that stabilizing and maintaining the number of surface AMPARs may potentially be useful in the treatment of Alzheimer's disease. In the present study, we examined the roles of post-translational ubiquitination and phosphorylation on the GluA1 subunit in mediating Aβ-induced down-regulation of surface AMPARs in cultured neurons.

Our main results indicate that 5 μm Aβ directly promotes the ubiquitination of GluA1 after 1 h of treatment. In contrast, a recent study reported that 250 nm soluble Aβ secreted from Chinese hamster ovary cells overexpressing APP V717F does not cause any apparent increase in AMPAR ubiquitination after 14 h of incubation (17). We reason that this discrepancy is due to the low concentration of Aβ used in this study. Indeed, our results show that Aβ promotes GluA1 ubiquitination in a dose-dependent manner and elicits the maximum effect at 10 μm. However, the effect of a low concentration of Aβ could be unmasked by AMPA treatment. Consistent with our findings, this form of soluble Aβ enhanced the ligand-induced ubiquitination of AMPARs. Previous studies from our laboratory and others have demonstrated a role for GluA1 ubiquitination in regulating the activity-dependent trafficking of AMPARs (21–23). Although the role of GluA1 ubiquitination in AMPAR endocytosis remains controversial, it is clear that it regulates AMPAR postendocytic sorting into late endosomes for degradation (35, 36). Indeed, we recently demonstrated that GluA1 ubiquitin-deficient mutants (K868R and qKR) are inefficiently sorted into late endosomes and are instead recycled back to the plasma membrane (23). As a result, these mutant receptors are spared from ligand-induced degradation in lysosomes. Interestingly, these GluA1 ubiquitin-deficient mutants also inhibit Aβ-induced down-regulation of surface AMPARs, probably by promoting the recycling of internalized receptors back to the plasma membrane.

What is the mechanism that allows these GluA1 ubiquitin-deficient mutants to be sorted into recycling endosomes? One of the major signaling pathways that regulate the reinsertion of AMPARs via recycling endosomes is the PKA-dependent phosphorylation of GluA1 at Ser-845 (19, 20). Consistent with this model, we found that the level of Ser-845 phosphorylation induced by forskolin stimulation was significantly higher in the GluA1 K868R and qKR mutants compared with wild-type receptors. Interestingly, we also discovered that the GluA1 S845D phosphomimetic mutant results in a lower ubiquitination level due to reduced binding with the GluA1 E3 ligase Nedd4-1. Importantly, Aβ is known to induce the dephosphorylation of GluA1 within 1 h of treatment (11), the same time course that led to a rise in GluA1 ubiquitination in our study. The functional relevance of these findings is underscored by the fact that GluA1 S845D phenocopies the effects of GluA1 ubiquitin-deficient mutants in preventing Aβ-induced removal of surface AMPARs. Furthermore, these results also help to explain why the GluA1 S845D phosphomimetic mutant is generally more stable and less prone to lysosomal degradation (29, 30). Overall, our findings describe, for the first time, an inverse relationship between GluA1 Ser-845 phosphorylation and ubiquitination in neurons. Such cross-modulatory regulation ensures the correct sorting and targeting of AMPARs in maintaining synaptic transmission and plasticity. We propose that these mechanisms may be hijacked by the neurotoxin Aβ during the pathogenesis of Alzheimer's disease, causing the dysregulation of AMPAR sorting and persistent loss of surface receptors that eventually lead to synaptic depression.

**Figure 7.**
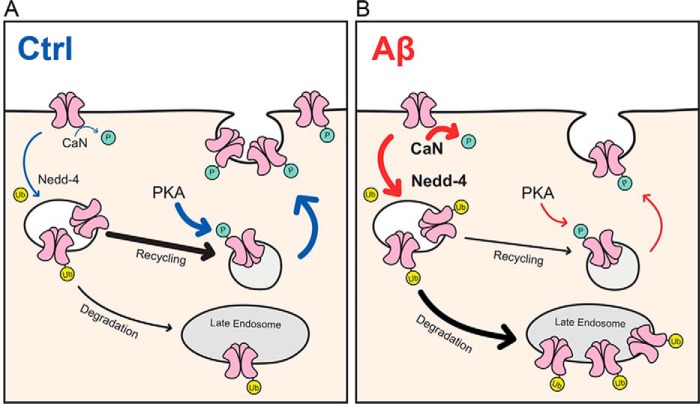
**Proposed model for the role of protein ubiquitination in mediating Aβ-induced down-regulation of surface AMPARs.** *A*, under normal conditions, the majority of internalized AMPARs are recycled back to the plasma membrane, a process that is facilitated by PKA phosphorylation of the GluA1 subunit at Ser-845. The phosphorylation of GluA1 prevents the ubiquitination of AMPARs as a result of which receptor degradation is maintained at a low level for normal protein homeostasis. *B*, however, in the presence of high concentrations of soluble oligomeric Aβ, AMPARs are constantly endocytosed due to overactivation of the protein phosphatase calcineurin (*CaN*) and the E3 ubiquitin ligase Nedd4-1. This in turn leads to an increase in the level of GluA1 ubiquitination in part due to the down-regulation of Ser-845 phosphorylation. As a consequence, the recycling of AMPARs back to the plasma membrane (and therefore their expression on the cell surface) decreases, leading to synaptic depression. *Ctrl*, control; *Ub*, ubiquitin.

In conclusion, our study has provided strong evidence for a direct role of GluA1 ubiquitination in mediating the adverse effect of Aβ in reducing the number of surface AMPARs. Based on our results, we propose the following working model (Fig. 7). Under basal conditions, most AMPARs are recycled back to the plasma membrane, a process that is mediated by PKA phosphorylation of the GluA1 subunit at Ser-845. The phosphorylation of GluA1 ensures that the ubiquitination of AMPARs occurs at a low level to maintain the normal protein homeostasis of the receptors. However, in the presence of high concentrations of soluble oligomeric Aβ, AMPARs are constantly endocytosed. In addition, the level of GluA1 ubiquitination increases in part due to the activation of the Nedd4-1 protein (17) and the protein phosphatase calcineurin (11, 31). The latter leads to down-regulation of Ser-845 phosphorylation, which reduces the sorting of AMPARs into recycling endosomes. As a consequence of AMPAR missorting, the recycling of AMPARs back to the plasma membrane is impaired, and thus their expression on the cell surface decreases, leading to synaptic depression. In a broader context, our data provide new mechanistic insights into how cross-modulation of GluA1 phosphorylation and ubiquitination can fine-tune the dynamics of AMPAR intracellular trafficking and the sorting decision that ultimately determines the number of receptors on the cell surface. Future studies will focus on GluA1 K868R knock-in mice, which would allow us to confirm the role of Aβ-induced AMPAR ubiquitination in mediating synaptic depression and spine loss in an Alzheimer's mouse model, without the need to overexpress exogenous GluA1 mutants (36).

## Experimental procedures

### Materials

The plasmids encoding full-length wild-type pH-GluA1 and the K868R and qKR mutants have been described previously (23). The pH-sensitive GFP (pHluorin) tag was inserted after the signal peptide sequence in the extracellular N-terminal region of GluA1. C-terminal serine to alanine or aspartate mutants for pH-GluA1 were generated using the standard overlap extension polymerase chain reaction protocol.

Specific antibodies against GluA1 (4.9D; 1:5,000) and GFP (JH4030; 1:1,000) were generated in the Huganir laboratory and have been characterized previously (23). The following antibodies were purchased from commercial sources: anti-ubiquitin clone P4D1 (Santa Cruz Biotechnology; 1:1,000) and clone FK2 (Enzo Life Sciences), anti-GluA1 phospho-Ser-831 (Millipore, AB5847; 1:2,500), anti-GluA1 phospho-Ser-845 (Millipore, AB5849; 1:2,500), anti-HA clone C29F4 (Cell Signaling Technology; 1:1,000), anti-synaptophysin clone 7.2 (Synaptic System; 1:10,000), and anti-β-actin clone C4 (Millipore; 1:10,000).

### Neuronal culture and electroporation

Primary cortical neurons were prepared from E18 rat pups as described previously (37, 38). They were plated onto poly-l-lysine-coated dishes or coverslips in Neurobasal growth medium supplemented with 2% B27, 2 mm GlutaMAX, 50 units/ml penicillin, 50 μg/ml streptomycin, and 5% fetal bovine serum (FBS). The neurons were switched to 1% FBS Neurobasal medium 24 h postseeding and fed twice a week. Cortical neurons were electroporated at DIV0 prior to plating using the Amaxa Nucleofector II system (Lonza) and were used at DIV13.

### Treatments

HPLC-purified Aβ(1–42) peptides were synthesized by Dr. James Elliott (Yale University). To prepare Aβ for treatment, peptides were dissolved in DMSO (5 mm), diluted in F-12 medium (Invitrogen) as a 100 μm stock solution, and incubated at 4 °C overnight. Following centrifugation, soluble Aβ was used to treat neurons at 5 μm final concentration for 1 h at 37 °C. Aβ solution was made fresh for every experiment. To induce GluA1 phosphorylation at Ser-831 and Ser-845, neurons were treated with PMA (Sigma; 0.1 μm) and forskolin (Sigma; 1 μm), respectively, for 10 min.

### Ubiquitination assay

The ubiquitination of AMPARs was induced by incubating neurons in artificial cerebrospinal fluid (ACSF; 25 mm HEPES, 120 mm NaCl, 5 mm KCl, 2 mm CaCl_2_, 2 mm MgCl_2_, 30 mm d-glucose, pH 7.4) containing 50 μm AMPA (Tocris), 50 μm d-2-amino-5-phosphonovaleric acid (Tocris), and 1 μm tetrodotoxin (Tocris) for 10 min at 37 °C. Neurons were then lysed in warm 1% SDS (in PBS) and diluted in 10 volumes of ice-cold cell lysis buffer (1% Triton X-100, 1 mm EDTA, 1 mm EGTA, 50 mm NaF, 5 mm sodium pyrophosphate in PBS) supplemented with 10 mm *N*-ethylmaleimide and Complete EDTA-free protease inhibitor mixture (Roche Applied Science). Lysates were centrifuged at 14,000 rpm for 20 min at 4 °C and cleared with protein A- or G-Sepharose beads. Precleared lysates were then incubated with antibodies (anti-ubiquitin, anti-GluA1, or anti-GFP) coupled to protein A- or G-Sepharose overnight at 4 °C followed by four washes with ice-cold lysis buffer and elution in 2× SDS sample buffer. The immunoprecipitated proteins were resolved by SDS-PAGE and probed by Western blot analysis with specific antibodies against GluA1 and ubiquitin.

### Surface biotinylation assay

The chemical labeling of surface proteins with biotin was performed as described previously (39). Briefly, neurons were washed twice with ACSF and incubated with 0.5 mg/ml Sulfo-NHS-SS-Biotin (Pierce) for 30 min on ice. Free biotin was quenched by washing cells twice with ice-cold 50 mm glycine, pH 7.4 (in ACSF). Cultures were lysed and sonicated in radioimmune precipitation assay buffer (1% Triton X-100, 0.5% sodium deoxycholate, 0.1% SDS, 2 mm EDTA, 2 mm EGTA, 50 mm NaF, 10 mm sodium pyrophosphate in Tris-buffered saline) and incubated with NeutrAvidin beads (Pierce) for 3 h at 4 °C. Beads were washed three times and eluted with 2× SDS sample buffer followed by Western blot analyses.

### Surface staining assay

The immunolabeling of surface pH-GluA1 with anti-GFP antibody was performed as described previously (37). Briefly, cultured cortical neurons expressing pH-GluA1 (either wild type or K868R or qKR mutant) were washed twice with ACSF and incubated with rabbit anti-GFP antibody for 30 min at 4 °C. Neurons were subsequently fixed with 4% paraformaldehyde, 4% sucrose solution (in PBS); permeabilized with 0.25% Triton X-100 solution (in PBS); and blocked in 10% normal goat serum. Neurons were then incubated with mouse anti-synaptophysin antibody at 4 °C overnight. The surface pH-GluA1 and synaptophysin were visualized by Alexa Fluor 568-conjugated anti-rabbit and Alexa Fluor 647-conjugated anti-mouse secondary antibodies. Endogenous GFP signals were used to determine the total expression of pH-GluA1 in neurons. Images were collected with a 63× oil-immersion objective on a Zeiss Axio Imager fluorescence microscope. Fluorescence intensities were quantified using ImageJ software (National Institutes of Health) for synaptophysin and surface and total pH-GluA1. Data were expressed as the surface/total pH-GluA1 ratio and normalized to their respective vehicle (DMSO) control in each groups.

### Co-immunoprecipitation assay

HEK293 cells were grown in DMEM supplemented with 10% FBS, 2 mm GlutaMAX, 50 units/ml penicillin, and 50 μg/ml streptomycin. Cells were transfected using the calcium phosphate precipitation method and lysed 48 h later with ice-cold cell lysis buffer supplemented with 10 mm *N*-ethylmaleimide and Complete EDTA-free protease inhibitor mixture. Cell lysates were centrifuged at 14,000 rpm for 20 min at 4 °C and cleared with protein A-Sepharose beads. Precleared lysates were then incubated with antibodies coupled to protein A-Sepharose overnight at 4 °C followed by four washes with ice-cold lysis buffer and elution in 2× SDS sample buffer. The immunoprecipitated proteins were resolved by SDS-PAGE and visualized by Western blot analysis.

## Author contributions

S. G. conducted most of the experiments. S. E. J. prepared primary neuronal cultures. T. Z. performed the GluA1-Nedd4-1 co-immunoprecipitation assay in HEK293 cells. R. L. H. contributed molecular reagents. J. W. designed research and conducted experiments in Fig. 4. V. A. conceived the project, designed research, and wrote the manuscript. All authors analyzed the results and approved the final version of the manuscript.

## Footnotes

4 The abbreviations used are:

Aβ: amyloid-β
ACSF: artificial cerebrospinal fluid
AMPAR: AMPA receptor
DIV: days *in vitro*
PMA: phorbol 12-myristate 13-acetate
APP: amyloid precursor protein
pH-GluA1: pHluorin (pH-sensitive GFP)-tagged GluA1
qKR: K813R/K819R/K822R/K868R
ANOVA: analysis of variance.
